# Disparities in access to ear and hearing care in Cambodia: a mixed methods study on patient experiences

**DOI:** 10.1017/S0022215122001396

**Published:** 2023-04

**Authors:** C J Waterworth, C T M Watters, T Sokdavy, P L Annear, R Dowell, C E Grimes, M F Bhutta

**Affiliations:** 1 Department of Audiology and Speech Pathology, University of Melbourne, Melbourne, Australia; 2 Nossal Institute for Global Health, University of Melbourne, Melbourne, Australia; 3 Guy's and St Thomas’ NHS Foundation Trust, London, UK; 4 Children's Surgical Centre, Kien Khleang Rehabilitation Centre, Phnom Penh, Cambodia; 5 King's Centre for Global Health and Health Partnerships, School of Population Health and Environmental Sciences, King's College London, London, UK; 6 Clinical and Experimental Medicine, Brighton & Sussex Medical School, Brighton, UK; 7 Department of ENT, Brighton and Sussex University Hospitals NHS Trust, Brighton, UK

**Keywords:** Hearing Loss, Otolaryngology, Health Services Accessibility, Health Policy, Cambodia

## Abstract

**Objective:**

Chronic suppurative otitis media is a major global disease disproportionately affecting low- and middle-income countries, but few studies have explored access to care for those with ear and hearing disorders.

**Method:**

In a tertiary hospital in Cambodia providing specialist ear services, a mixed method study was undertaken. This study had three arms: (1) quantitative analysis of patients undergoing ear surgery, (2) a questionnaire survey and (3) semi-structured in-depth interviews.

**Results:**

Patients presented with advanced middle-ear disease and associated hearing loss at rates that are amongst the highest per capita levels globally. Patients reported several structural, financial and socio-cultural barriers to treatment. This study showed a significant burden of ear disease in Cambodia, which reflects a delay in receiving timely and effective treatment.

**Conclusion:**

This study highlights the opportunity to integrate effective ear and hearing care into primary care service provision, strengthening the package of activities delivered at government facilities.

## Introduction

Ear disease and hearing loss have widespread and significant implications on a person's quality of life, education, socio-economic opportunity and well-being. The economic cost of hearing loss on a global scale is enormous, with recent conservative estimates of nearly 1 trillion US dollars annually.^1^ There is a significant inequality in the distribution of hearing loss, with 80 per cent of the global burden impacting those residing in low- and middle-income countries.^2^

Chronic suppurative otitis media (CSOM) is a major global disease, disproportionately affecting those in low- and middle-income countries.^3^ CSOM is characterised by a perforation of the ear drum, with or without the presence of a cholesteatoma, intermittent or continuous ear discharge, and around two-thirds of patients experience a moderate or worse hearing loss.^3^ Medical treatment of ear discharge in CSOM typically includes topical antibiotic drops^4^ and monitoring, but long-term resolution often necessitates surgery via repair of the ear drum, termed tympanoplasty, which often improves hearing, or excision of the cholesteatoma, termed mastoidectomy, to safely remove the disease.

In many low- and middle-income countries, patients residing in rural and semi-rural settings frequently present with advanced disease or complications,^5^ which are compounded by the interplay between impoverishment and the inequitable distribution of ear and hearing care services.^6^ In fact, within low- and middle-income countries, chronic ear discharge is one of the most common reasons for seeking specialist ear care services.^7^

Improving access to healthcare in low- and middle-income countries has been a long-term and ongoing concern for researchers and policy makers, yet significant disparities continue to exist.^8^ There is a body of literature on minimising the barriers to accessing care in low- and middle-income countries^9^^,^^10^ and on the effectiveness of interventions designed to improve access to care.^11^ Interventions designed to expand healthcare access for poor and disadvantaged communities are evident in a number of South Asian countries^12^ and in Southeast Asia. In Cambodia for example, Liverani *et al*. (2017) highlighted the complex and contextual barriers to improving access to treatment for malaria in remote areas of Kampot.^13^

Southeast Asia has one of the highest prevalence rates of CSOM in the world,^14^ yet there is very little literature regarding the state of ear and hearing care in Cambodia. To date, there have been no nationally recognised prevalence studies on hearing loss or ear disease. A population-based, cross-sectional national survey conducted between 2011 and 2012 reported hearing loss as the most common impairment amongst children, with 6.53 per cent of children recorded as having a disability.^15^ In 2013, it was reported that less than 2000 of the estimated 51 000 profoundly deaf Cambodians had access to deaf services.^16^ More recently, in 2016, the World Health Organization described recurrent ear discharge as being normalised amongst rural children.^17^ Gaps in the literature remain in terms of understanding the magnitude and impact of ear disease in the country, the supply of ear and hearing care providers including otolaryngologists and audiologists, and the key challenges that Cambodian people face in accessing timely and appropriate health and rehabilitation services relative to the need. Exploring access to receiving ear care is of particular policy relevance in Cambodia, where there is a lack of evidence on the severity and impact of ear disease, a significant inequity in available services, especially in rural areas, and an ongoing challenge in incorporating ear care into government policy and planning.

Several studies have explored challenges to the provision of medical^8^ and surgical^18^ care in low- and middle-income countries, but very few have explored the experience of those with chronic ear disease in their journey to seek ear and hearing care services.

The purpose of this study was to examine the experience of a cohort of patients who presented at Cambodia's principal ear care hospital as they sought care for their ear conditions. We investigated the severity of their disease and its impact, and we made an analysis of their healthcare-seeking for diagnosis and treatment, including delays in accessing care, the severity of the disease, distance to care, and the nature of ear and hearing care service delivery.

### Background

Cambodia, now classified as a lower middle-income country,^19^^,^^20^ has achieved strong economic growth and has pioneered social health protection programmes designed to improve access to healthcare for the poor. In the last 20 years, there has been a significant decrease in the official poverty rate to 12.9 per cent of the population by 2018.^15^^,^^21^ The effective level of poverty, however, is much higher, and significant inequalities in standards of living between rural and urban areas prevail.^22^ Under the government's health coverage plan, health facilities (hospitals and health centres) have been placed equally across the country according to uniform population catchment areas. However, access to quality care remains a challenge, particularly for impoverished Cambodians residing in rural areas.^23^ Concerns about financial support for diagnosis and management of non-communicable diseases remain a challenge and are currently a focus of national health planning.^24^^,^^25^

The Cambodian health system consists of public and private providers. The public sector has undergone dramatic change since 1996, when nominal user fees were introduced at government health facilities, to increase access to services and improve healthcare coverage.^26^ Over the last 20 years, the private sector has expanded but remains largely unregulated, with a mixture of both qualified providers working in health facilities and unqualified providers, such as traditional healers and merchants, selling medications and offering services.^24^^,^^25^ Approximately 60 per cent of total health expenditure consists of household out-of-pocket payments, which are directed principally to unregulated private providers.^27^ In rural areas, only 15 per cent of primary care occurs in the public sector, and private, non-medical (unqualified) providers account for half of all healthcare providers.^28^

The Ministry of Health has committed to universal health coverage and has endeavoured to achieve more equitable access to care through its consecutive health strategic plans, the most recent being the Third Health Strategic Plan 2016–2020.^29^ Currently, public health facilities deliver services through 24 provincial health departments, which operate a provincial hospital in each province and govern 81 operational districts.^27^ Within operational districts, a referral hospital delivers the complimentary package of activities covering secondary level care, and health centres provide the minimum package of activities covering prevention and basic treatment.^27^

However, very few government hospitals or health centres provide ear and hearing care services, which are mostly provided by non-government organisations with foreign connections, primarily in Phnom Penh with a few others scattered throughout the country.^30^ In Phnom Penh, some public and private hospitals have established ENT departments. Among the government hospitals are Preah Ang Duong Hospital, the Khmer–Soviet Friendship Hospital, the National Paediatric Hospital, Calmette Hospital and Preah Kossamak Hospital.

Among non-governmental organisation supported facilities, the Children's Surgical Centre in Phnom Penh is a major provider of ENT services. All Ears Cambodia, a non-governmental organisation that has five clinics across the country, works with government and non-government service providers to increase coverage and awareness of primary ear care and is building a local workforce of ear and hearing care health workers.^15^ Special education is available for a limited number of children with hearing loss, who can attend Krousar Thmey School (preparation year 12), a local non-governmental organisation school that was officially transferred to the Ministry of Education, Youth and Sports in 2019, and young adults (16 years or over) with profound hearing loss who can attend the non-governmental organisation supported Maryknoll Deaf Development Program, which provides vocational training, Cambodian sign language training and basic education.^16^^,^^31^

The Children's Surgical Centre, a non-governmental organisation charity hospital in Phnom Penh, has been the country's main provider of surgical ear care since 2014, following a sustained in-country training programme by UK-trained surgeons.^32^ The Children's Surgical Centre provides free surgery and treatment to impoverished adults and children, covering all in-hospital expenses except for food, transportation or accommodation outside of the hospital. The ENT department primarily focuses on treatment and rehabilitation of CSOM (including cholesteatoma), with approximately 250 tympanoplasty or mastoidectomy operations performed each year.

## Materials and methods

We set out to answer a number of key research questions: What is the extent of ear disease in Cambodia? What are the patterns of utilisation of ear care services? What challenges do people face in obtaining appropriate ear care, and how can these challenges be addressed? Measuring access to care has been deemed a challenging task, but it ultimately relies on the ability to assess whether the characteristics of services, providers and systems are aligned with people, households and community capabilities.^33^

Barriers to care may be structural, financial or socio-cultural,^34^ which Levesque *et al*. (2013) conceptualised into a framework that encapsulates the demand and supply determinants that disrupt or delay the healthcare-seeking journey.^33^ This is a widely accepted health system framework that incorporates both health providers’ and healthcare users’ perspectives on access and has been used previously to explore, assess and measure access in various healthcare services and settings.^35^ Structural barriers include location of facilities, transportation, childcare and long waiting times, socio-cultural barriers include lack of knowledge or acceptance among local communities, and financial barriers include a lack of, or inadequate, health protection schemes. We adopted the Levesque framework for assessing access to ear and hearing care.

We employed a mixed methods approach to provide a better understanding of the multi-faceted challenges to ear and hearing care access through the triangulation of the data. Three data collection strategies were adopted: a quantitative analysis of the Children's Surgical Centre patient database to establish the extent of ear disease presenting to the hospital; a patient survey to establish a broad overview of the main challenges experienced; and in-depth qualitative interviews of patients and providers to gain further insights into the important factors hampering access to ear and hearing care of those attending the hospital for treatment.

For the extraction of data on symptomology, markers of disease severity and correlation with markers of disease severity to distance from hospital, we obtained electronic records of patients who underwent tympanoplasty surgery (for CSOM) or mastoidectomy surgery (for cholesteatoma) at the Children's Surgical Centre between October 2014 and April 2019 (excluding records for second-side surgery).

We extracted data on markers of disease severity, specifically: (1) symptoms and their duration at presentation; (2) mean air conduction hearing thresholds in decibels in the worse ear on pre-operative pure tone audiometry at 500 Hz, 1000 Hz, 2000 Hz and 4000 Hz; and (3) grading of anatomical destruction of temporal bone structures according to data from operative records.

For patients undergoing tympanoplasty, we scored size of tympanic perforation as: less than 30 per cent of the tympanic membrane = 1 point; 30–60 per cent of the tympanic membrane = 2 points; and more than 60 per cent of the tympanic membrane = 3 points. We added 1 more point if erosion of ossicles was present. We classified patients as: 1 point = grade 1; 2 points = grade 2, and equal to or more than 3 points = grade 3.

For patients undergoing mastoidectomy, we summed the number of temporal bone structures eroded from the following list: malleus, incus, stapes, chorda tympani, external ear canal, facial canal, tegmen, bone over posterior fossa and lateral semi-circular canal. We classified patients: equal to or less than 2 structures eroded = grade 1; 3–5 structures eroded = grade 2, and equal to or more than 6 structures eroded = grade 3.

We used patient records to explore the relationship between travel distance to the hospital, delays in accessing care and the severity of ear disease. For each patient, we used Google Maps (Google, Mountains View, USA) to estimate distance (in kilometres) and travel time (in minutes) to the Children's Surgical Centre from their commune of residence. Patient data were collated, anonymised and exported to Minitab 19® statistical software. For mean pure tone threshold and symptom duration (continuous data), we performed Pearson linear correlation to distance travelled. For grade of anatomical destruction (ordinal data), we compared distance travelled in each group using Tukey pairwise comparison.

Over a six-month continuous period (March to September 2019), we invited adults or primary caregivers of children with ear or hearing symptoms attending the Children's Surgical Centre ENT Department to complete a questionnaire on their journey prior to attending the hospital (Appendices 1–3). Survey questions were translated into Khmer and translated back into English for verification prior to interviews. Because of the varying literacy of participants, questionnaires were completed by local nurses in Khmer language under the local supervision of the head of the ENT department and researcher (TS). Prior to formal data collection, the questionnaire was tested amongst a group of Khmer-speaking patients and staff members to establish its reliability and to check for ambiguity; this group predominantly, but not exclusively, suffered from CSOM. We asked about participant or patient experiences of disease and the journey leading them to hospital. A power analysis in G*Power 3.1.9.2 statistical power analysis software determined a target sample size of 114 participants (alpha, 0.05; power, 0.95) for detecting a correlation between two numerical variables of medium effect (*r* > 0.30).^36^

Through patient records and the questionnaire survey, we purposively selected 15 adults and 5 caregivers to participate in semi-structured interviews to gain more understanding into the lived experience of people with ear disease and hearing loss who were seeking out ear and hearing care services. Interviews were conducted in August and September 2019 in a private room at a café that was a short walking distance from the Children's Surgical Centre hospital by two English-language researchers assisted by two local Khmer interpreters. In order to ensure reflexivity, cultural competency and a healthy dialogue within the research team, pilot interviews were conducted to review the topic and discuss potential sources of bias and interpretation of data. We asked participants open-ended questions on topics related to health and ear health experiences, access, utilisation, demand for services, perception of quality of care and health beliefs. Each interview was audio recorded, transcribed verbatim (excluding Khmer), and content-coded and analysed using NVivo qualitative data analysis software (version 12, QSR International, Melbourne, Australia).

We used a deductive approach to analyse data, and categorised responses into the model devised by Levesque *et al*.^33^: approachability and ability to perceive need for care; acceptability and ability to seek care; availability and ability to reach care; affordability and ability to pay for care; and appropriateness and ability to engage with healthcare services. Codes were inductively derived from interview responses, compiled into categories and then merged into main determinants.

## Results

For the patient data, 693 records were identified in the study period of which 113 were excluded because of duplication or second-site surgery. We had data for 407 patients undergoing tympanoplasty (173 of 407 (43 per cent) male) of which 122 were children (age range, 6–17 years) and 285 were adults (age range, 18–61 years), and 173 patients undergoing mastoidectomy (85 of 147 (49 per cent) male) of which 44 were children (age range, 4–17 years) and 129 were adults (age range, 18–57 years).

Regarding symptoms at presentation (Table 1), in the tympanoplasty group the most common symptom was self-reported hearing loss (73 per cent), and in the mastoidectomy group it was otorrhoea (98 per cent), followed by self-reported hearing loss (78 per cent). Mean symptom duration prior to receiving formal ear care at the hospital was 13.5 years (± 11.7 standard deviation (SD)) for the tympanoplasty and 13.9 years (± 11.0 SD) for the mastoidectomy group, with no significant difference between groups (two-sample *t*-test, *p* = 0.697). Pre-operative pure tone audiometry results were available for 97 per cent (564 of 580) of patients. Mean thresholds in the worst ear were 46 dB HL (± 16.3 SD) for the tympanoplasty group and 60.2 dB HL (± 23.3 SD) for the mastoidectomy group, which was a significant difference (two-sample *t*-test, *p <* 0.001). Half of the tympanoplasty group (206 of 407 (51 per cent)) and 29 per cent (50 of 173) of the mastoidectomy group had an abnormal contralateral ear.

**Table 1.** Recorded symptoms at presentation for tympanoplasty and mastoidectomy cases in quantitative analysis

Parameter	Tympanoplasty* (*n* (%))	Mastoidectomy^†^ (*n* (%))
Otorrhoea	185 (45)	170 (98)
Self-reported hearing loss	296 (73)	135 (78)
Tinnitus	101 (25)	35 (20)
Otalgia	44 (11)	30 (17)
Vertigo	11 (2)	12 (7)

*n = 407; ^†^*n =* 173

Regarding anatomical markers of disease severity in the 407 patients undergoing tympanoplasty, 7 per cent (30) had ossicular erosion recorded, and 98 per cent (397) had a perforation, of which 11 per cent (43 of 397) had a small perforation (less than 30 per cent of the tympanic membrane), 48 per cent (191 of 397) had a medium perforation (30–60 per cent) and 41 per cent (163 of 397) had a large perforation (more than 60 per cent). Based on the grading system used, 47 tympanoplasty patients were grade 1, 190 were grade 2 and 166 were grade 3 (the most severe). In the 173 patients undergoing mastoidectomy, there was erosion of temporal bone structures in the following numbers: incus, 129 (75 per cent), stapes, 88 (51 per cent), malleus, 77 (45 per cent), chorda tympani, 39 (23 per cent), facial canal, 39 (23 per cent), lateral semi-circular canal, 16 (9 per cent), external ear canal, 14 (8 per cent), tegmen, 12 (7 per cent) and posterior fossa, 8 (5 per cent). Based on the grading system, 103 mastoidectomy patients were grade 1, 60 were grade 2 and 10 were grade 3.

A total of 114 patients participated in the questionnaire survey, including 89 adults (mean age ± SD, 37.6, ± 13.4 years; 45 of 89 (52 per cent) male) and 25 caregivers of children (mean age ± SD, 10.8 ± 3.6; 16 of 25 (64 per cent) male). Some questions were skipped by some participants because of time constraints. Table 2 summarises the challenges that participants experienced prior to attending the Children's Surgical Centre for ear care services. There was a relatively even distribution of supply and demand influences. Reasons for attending the Children's Surgical Centre were recorded in 114 responses, with the most common being worsening symptoms (111 of 114; 97 per cent) and recent knowledge of the services at the Children's Surgical Centre (105 of 114; 92 per cent). A high proportion (85 of 114; 75 per cent) also reported a lack of successful prior treatment as a reason to attend, and more than half (62 of 114; 54 per cent) attended because their ear condition was impeding their ability to work. Other reasons cited were because other hospitals do not treat ears (52 of 114; 46 per cent) or had too long a waiting time (25 of 114; 22 per cent), because they were referred by another health professional (14 of 114; 12 per cent) or because there was a change in circumstances (7 of 114; 6 per cent).

**Table 2.** Difficulties experienced seeking ear care prior to the Children's Surgical Centre*

Supply or demand	Reason	Value (*n* (%))
Supply/demand	Service location too far from house	81 (75)
Demand	Fear of treatment	68 (63)
Demand	No awareness of health service information	62 (57)
Demand	No education on ear health	54 (50)
Supply	Accommodation unavailable	47 (44)
Demand	Fear of hospital	44 (40)
Supply	Treatment or service expenses	43 (40)
Supply	Roads too poor	43 (40)
Supply	Transport expenses	38 (35)
Supply	Waiting list long	35 (32)
Demand	Could not miss work	26 (24)
Supply	Food expenses	13 (12)

* *n =* 108. Values taken from questionnaire

We also conducted semi-structured interviews with 15 adults (age range, 20–72 years, 8 male) and 5 caregivers of children (age range, 9–18 years, 2 male). Here we present the combined results of the questionnaire and interviews thematically, following the categories created by Levesque *et al*. (2013); specific patient quotations and interview excerpts related to each theme are shown in Table 3.

**Table 3.** Emergent themes from patient interviews

Dimension of access	Sub-theme	Excerpt
Approachability/ability to perceive need for care	Lack of knowledge or recognition of symptoms	‘So, it's because of her understanding. At that time, she thought [the ear problem] was not important. She can still go to school, move around, walk, can do everything … It's just smelling and discharge…’ (participant 4)
	Acceptance of traditional health beliefs	‘When he was a young baby, he cried a lot, so the tear is drop into the ear and he got infected in there’ (participant 10)
	Preference for traditional treatments or self-treatment	‘His son has a hearing problem, so he tried to find the treatment somewhere … people gave advice to get treatment from the traditional healer, but most of that treatment [did] not help much’ (caregiver 18)
	Practice of medical pluralism	‘She went to the doctor, but also asked help from the tree [spirit-belief/Kru Khmer], like combined together … she believes more in the doctor, but she likes to combine [treatment] together’ (caregiver 2)
	Transiency of symptoms	‘It [discharge] usually happens every one or two months, maybe five days each time, and then it gets better and then another month it goes away’ (participant 4)
Acceptability/ability to seek care	Fear or lack of trust in provider	‘Initially, she felt very, very, very scared. She felt not too confident with the hospital’ (participant 5)
	Fear of surgery	‘His family worry about after the surgery … Cannot work and affect his life after the surgery’ (participant 17)
	Fear of anaesthesia	‘He's scared about the operation because most Khmer people don't understand about the anaesthesia. They usually hear from other rumours, “the anaesthesia can make people die!”’ (participant 11)
	Stigma	‘She never told anybody. So scared. Scared to let everybody know her disease … She didn't want anybody to know she had the problem because the ear gets smell and dirty. The pus came out, so she tried to clean and keep secret. She doesn't want anybody to know her problem. In family is ok but [not] for everybody around’ (caregiver 8)
	Cultural or family influence on decision making	‘Everyone in her family influence her, like push her, to find treatment. Some people that know the place to go for treatment, they tell her’ (participant 1)
	Collectivism	‘It's still a problem [her mother's ear condition], so that's why her daughter stopped studying and helped her … she need to stop her dream to take care of her mother. Her dream is, she want to have cafe and bakery shop, but she need to stop everything to take care of her… Sometimes [older parents] try to keep this [information] by themselves. They don't tell anybody except husband or wife. They don't want the children to know. Cambodian children worry very much if they heard that their parents have any problem. If she saw her mum was sick, [her daughter would] decide to stop studying, [so] the parents decided to keep things [secret]’ (caregiver 8)
Availability/ability to reach care	Lack of available ear and hearing care services close by	‘The ear service around the commune, it's just simple general medical care, but no specialists’ (participant 7). ‘They said they don't have the ENT Department in their hospital, so they tell her to go to find the treatment outside, like a private clinic’ (caregiver 3)
	Lack of ability to navigate to service	‘His village is far away from the town [Phnom Penh]. He doesn't like to stay there and then it's difficult traffic and roads’ (participant 13)
	Occupational flexibility/seasonal factors	‘Right now [in] his neighbourhood, they plan to come to hospital to check, but you know the farmers have to decide the time. They need to be free from their farm work’ (participant 13)
	Lack of childcare	‘She has many children. It's hard to take time to bring the child with the problem to the hospital. She has a problem with money too. That's why [it's] difficult to bring the child to see the doctor’ (caregiver 15)
Affordability/ability to pay for care	Direct costs too high	‘They have to spend each time at least 40 to 50 USD each consultation and the medication, but her daughter looks not better. So, he decide to bring her to ‘free’ hospital’ (participant 3)
	Indirect (opportunity) costs too high	‘When he cannot hear well, it's difficult; it's hard to work … when he got sick, he difficult to earn money and then not enough money is difficult to find treatment’ (participant 12) ‘In his family, just only him that earn the money… so he is very important in the family. He cannot spend the time to get operation’ (participant 16)
Appropriateness/ability to engage with providers	Lack of patient centred care	‘…some hospital outside when she has a question, they just only shout back. She went many times for medical treatment, and she tried to ask why [is there] no cure for her daughter. They said “It's the disease for follow up! Cannot get cure quick!”, but the answer is not polite’ (caregiver 3)
	Lack of belief in providers	‘Before, he felt they don't believe in medical staff who work at the commune because they don't work with experience about ear care. And then may need to be charged money a lot! So, both problems – the money and the technique or experience to try to make [healthcare] work’ (participant 13)
	Lack of belief in the integrity of the medical system	‘They (hospital providers) don't do the right way. They take the money and they want to take again and again. Not just one way. So that is the way that they are making money into the hospital. So, most of the patients, they don't like to go and see them’ (participant 11)
	Medical paternalism	‘No doctor told him anything about what is the problem that child has … He just got medical treatment, like injection and perfusion, but they didn't know what it is’ (caregiver 18)

USD = US dollars

### Approachability and ability to perceive need for care

An individual's ability to perceive the need for ear care services related to three main factors: awareness of the likely reasons for hearing loss, health beliefs and transiency of symptoms. Half the patients completing the questionnaire (54 of 108; 50 per cent) reported that their lack of knowledge of ear conditions was a barrier to obtaining prior care. Among the in-depth interviewees, several demonstrated misunderstanding about their ear condition and its severity. Hearing loss was sometimes interpreted as a mental health problem or lack of concentration rather than a condition of the ear. Some participants were stoic and perceived the problem as unimportant, trivialising symptoms. Health-seeking behaviour may be influenced by traditional health beliefs.^37^ Some participants believed their ear disease was caused by things such as tears falling into the ears, exposure to dirty water while swimming, excessive use of earphones or over-cleaning the ears. The transient and fluctuating nature of ear and hearing symptoms meant that participants’ perceived need for care changed over time. During periods with lighter symptom burden, patients would delay seeking healthcare, believing that symptoms could resolve without intervention.

### Acceptability and ability to seek care

Once participants became aware of their need for ear care, fear was a significant barrier in initially seeking care, with 63 per cent (68 of 108) of patients in the questionnaire reporting fear of the treatment or surgery itself, the hospital and local environment, the outcomes of surgery or stigma from their community. Some lacked trust in the integrity of healthcare providers because of prior experience. For example, some thought that private providers would ask patients to return multiple times for financial gain, without any apparent improvement in symptoms.

The local culture of communal living and collectivism both helped and hindered health-seeking behaviour. The majority of participants reported support from friends and family in attending the hospital, and most discovered the hospital through relatives or neighbours in their village who had previously had a positive experience. Families were very influential in a participant's consideration for seeking and accepting care. Collectivism is a characteristic reflected in Cambodia and other Asian societies, whereby adult children become primary caregivers of their aging parents, often attending medical appointments together. It can in turn become a barrier to seeking care, with some participants explaining they might hide symptoms from other family members in order to prevent worry or responsibility.

### Availability and ability to reach services

A lack of local ear care services was the most frequent difficulty experienced (81 of 108; 75 per cent), and at least half of participants responding to the questionnaire believed their lack of knowledge of service availability (62 of 108; 57 per cent) was a barrier to obtaining prior care. Thirteen participants (12 per cent) had no knowledge of local ear care services, and the same number had not previously attended any such services.

**Fig. 1.**
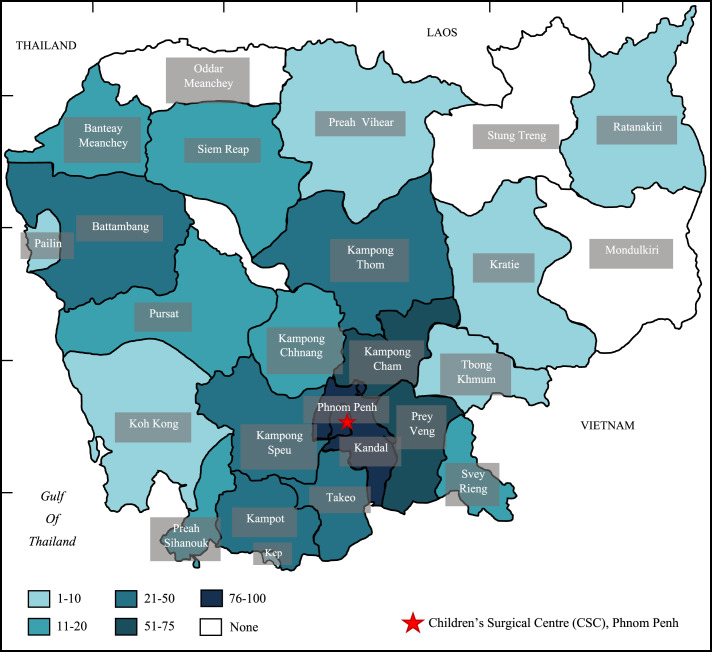
Distribution of Children's Surgical Centre patients across Cambodian provinces.

**Fig. 2.**
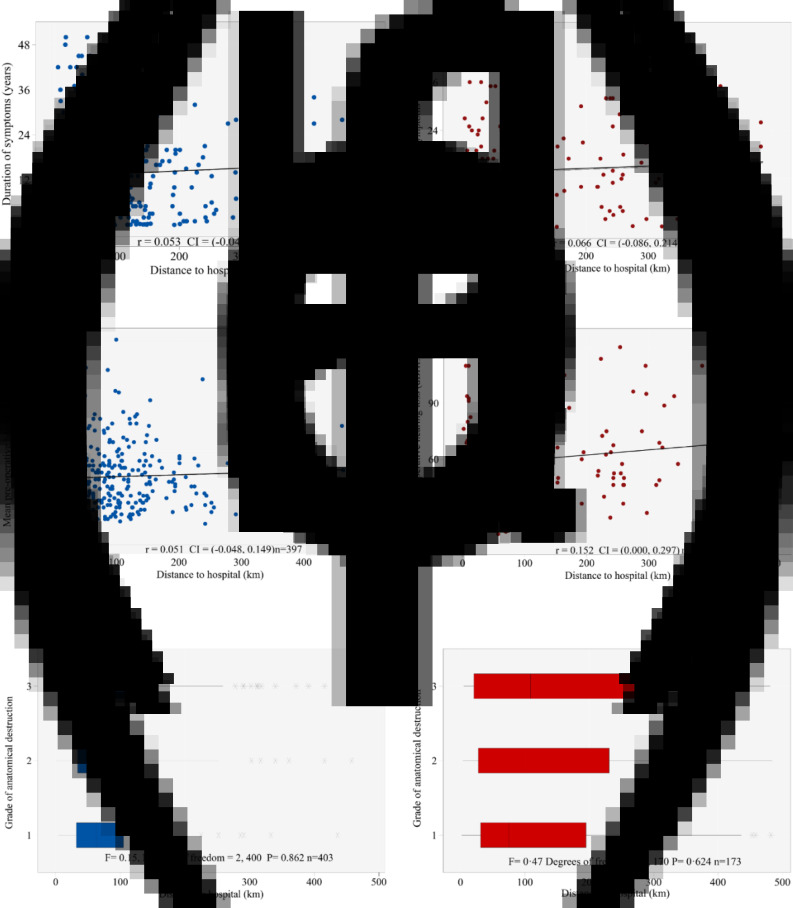
Distance to hospital in kilometres plotted against scatterplot of pre-operative hearing loss in cases of (a) tympanoplasty and (b) mastoidectomy cases. Scatterplot of duration of symptoms (years) in cases of (c) tympanoplasty and (d) mastoidectomy. Boxplot of grade of anatomical destruction in cases of (e) tympanoplasty and (f) mastoidectomy. CI = confidence interval

For participants living in rural or remote areas, accessing the Children's Surgical Centre (the main provider of free surgical ear care in the country) had also presented barriers, with some interviewees travelling up to eight hours via any transport method available to them (motorbike, taxi, minibus or public transport). Analysis of the quantitative data showed a large variation in distance and time travelled to the hospital (Figure 1), with a mean of 94 km (SD, 87.7; range, 0–463) and 124 minutes (SD, 89; range, 0–449) for the tympanoplasty group and 131 km (SD, 128.2; range 2–484) and 154 minutes (SD, 124; range 4–259) for the mastoidectomy group. Patients undergoing mastoidectomy travelled significantly further than those undergoing tympanoplasty (two-sample *t*-test for distance, *p* < 0.001; for time, *p* = 0.004). A total of 62 per cent (362 of 580) of patients lived within 100 km of the hospital. Correlation between distance travelled and markers of disease severity are shown in Figure 2; we found no significant correlation in any of these variables.

Where an ear care service was available in the home district, a similar proportion of utilisation to availability was observed (Table 4), suggesting that where services are available and known about, in general they were utilised. For those who did seek available modern medical services, they visited pharmacies, private medical clinics, public health centres, district health centres, and out-patient departments in government or non-government hospitals. Nonetheless, the majority recounted numerous barriers, particularly the lack of ear health specific services and treatments or uncertainty about how to navigate the health system in order to receive relevant and specific care.

**Table 4.** Patients’ perception of availability and utilisation of ear care services prior to attending the Children's Surgical Centre*

Type of provider	Service availability in participant's home district (*n* (%))	Service utilisation prior to Children's Surgical Centre (*n* (%))
Pharmacy	79 (70)	65 (58)
Private hospital/health centre	40 (35)	53 (47)
Government hospital/health centre	34 (30)	32 (28)
Specialist ear doctor	3 (3)	8 (7)
Traditional healer	5 (4)	5 (4)
No health service	13 (12)	13 (12)

* *n =* 113. Values taken from questionnaire

Information about prior treatments was recorded for 113 questionnaire respondents, with the majority receiving oral medication (93 of 113; 82 per cent) or ear drops (70 of 113; 62 per cent). A smaller proportion had received prior ear surgery (9), used traditional medicine (3) or treated the ear in some manner themselves (16). Eleven patients reported no prior treatment. Several participants reported previously using traditional home remedies, often passed down from older family members and widely accepted. This included placing various items into the ears, such as perfume, coconut oil, papaya oil, garlic, pepper, tobacco leaves and feathers. Many (from both urban and rural abodes) sought care from traditional healers (Kru Khmer) prior to attending the hospital.

Kru Khmer were employed to treat severe or chronic discharge, ear pain or hearing loss. For discharging ears, treatments included cleaning the ears with chicken feathers, pouring hot wax around the ears, chewing tree roots and spitting them into the ears, burning (cauterising) with burnt cotton buds or cigarettes, and smoking the ears with long pieces of wood inserted into the ear canal. For hearing loss, treatments included massaging or slapping the ears. In most cases, such treatments were reported to be unsuccessful, provide only temporary relief or sometimes cause additional issues. Some participants practised medical pluralism, using traditional and modern medicine in conjunction.

### Affordability and ability to pay

Most participants cited out-of-pocket expenditure as a barrier to accessing ear care, which was why many initially sought care from a local traditional healer or pharmacy prior to attending a formal healthcare facility. Mean out-of-pocket expense (for patients in the questionnaire) prior to attending the Children's Surgical Centre was US$614 for adults (range, $10–2000) and $575 for caregivers (range, $50–1000). For those accessing formal care, participants reported difficulties in raising funds; some were able to self-fund completely whereas most had to work extra hours, access savings, or borrow from relatives or friends. One participant recounted selling her jewellery to pay for her daughter's ear treatment, and another reported spending US$2000 throughout their ear care journey.

The inability to pay direct medical costs including hospital fees, surgical fees and medication expenses in addition to non-medical costs, such as transportation, food and accommodation, caused some participants to delay treatment. Indirect costs were represented by expenditures incurred beyond direct medical costs (such as transport), and income-earning or childcare roles prevented some from dedicating time to health needs (for example, farmers who were understandably reluctant to travel during the rice harvesting season given the potential impact on their livelihood).

### Appropriateness and ability to engage with providers

The respondents at the Children's Surgical Centre believed that they had previously received sub-optimal care, and this impacted their ability to engage fully with the service provider. Several participants reported concerns of a low-quality service at previous providers and a failure to be referred to specialist services. There were also reports of poor communication, lack of trust and paternalism from medical professionals. Many participants sought care at charitable organisations, such as the Children's Surgical Centre, when they became aware of this option and other treatments had not worked.

## Discussion

To our knowledge, there is no documented evidence on the prevalence and severity of ear disease or hearing loss in Cambodia. Although hearing loss has been ranked the fourth leading chronic disease globally, data are available across only 16.5 per cent of all geographic areas.^38^ Regarding CSOM specifically, many social factors reportedly contribute to the complexity of the disease, including impoverishment, marginalisation, malnutrition, a lack of quality health services, poor education and a lack of evidence-based treatment protocols.^39^ Compared with similar studies in other countries, our findings confirm that the degree of hearing loss and disease progression for the Children's Surgical Centre cohort is amongst the most severe reported globally for patients undergoing either tympanoplasty^40–46^ or mastoidectomy surgery.^41–43,47–51^ Patients presented to the Children's Surgical Centre on average 13–14 years after they initially became aware of their symptoms, a delay which likely contributed to the severity of the disease and degree of hearing loss. A high proportion of patients living geographically closer to the hospital experienced a similar severity of disease to those living remotely, which suggests that distance to services is not the most significant barrier to access but rather the general lack of availability of appropriate care.

The Children's Surgical Centre cohort experienced many of the typical supply- and demand-side challenges that affect access, including a lack of knowledge of ear disease or provider information, high direct and in-direct healthcare costs, opportunity costs and time lost because of long distances travelled (which our cohort ultimately overcame when accessing the Children's Surgical Centre). On the supply side, most participants reported a lack of ear and hearing care services at the primary care level and even fewer specialist services at the secondary or tertiary level, and they resorted instead to ‘simple medical care’. On the demand-side, the preference for private providers (qualified and informal) reflects a general belief that they provide better quality of care than government services.^37^ The common resort to self-treatment (visiting the local pharmacy, seeking advice from family and sometimes practising medical pluralism with Kru Khmer) mirrors findings from Rwanda, for example, where medical pluralism is more common in rural settings and where modern healthcare facilities may be unavailable.^52^

The lack of awareness of the causes of hearing problems and the lack of knowledge of ear and hearing care services, are likely to reflect the fact that services are simply not available locally, evidenced also in feelings of fear, stigma and a lack of trust in providers. This is also demonstrated by the fact that care was sought where it was locally available (demonstrated by prior treatments) and that patients were willing to pay high out-of-pocket costs (US$500–600 expenditure is high related to average incomes) to get care even in places where neither professional services nor quality care were available.

This preliminary scoping study shows a significant burden of ear disease in Cambodia that is related to the delay in receiving timely and effective treatment and that exceeds current workforce and infrastructural capacity in both public and private sectors. As healthcare facilities were generally unavailable in rural areas, people tended to ‘get by’, learning to live with their symptoms for a significant period of time. The lack of health facilities specialising in ear and hearing care, poor quality of care and ensuing worsening of their chronic symptoms, encouraged patients to continue their ‘health shopping' behaviour until they reached the Children's Surgical Centre hospital. Our findings reflect gaps in the knowledge of healthcare providers about causes and treatment of otitis media and a lack of co-ordination of care between providers within the health system.

The global evidence indicates that early treatment for CSOM involves inexpensive topical antibiotics and ear cleaning and has been reported to be managed by non-specialists in appropriately equipped local or regional health facilities.^5^ A model utilising community healthcare workers has been used to deliver ear and hearing care in other low resource settings,^53^^–^^55^ although the current structure to support such workers in Cambodia is somewhat fragmented.^56^ Community healthcare workers have been an essential link between health centres and the community and are seen as key for health education and promotion of prevention activities.^57^ Stigma and lack of understanding of disease requires education and cultural change, where again local or regional community engagement may be relevant as well as communication and policy change at the national level.

- Despite the reported high prevalence of ear disease in Southeast Asia, few studies have explored access to care for those with chronic suppurative otitis media
- Cambodia has seen significant improvements in health outcomes since the introduction of health financing policies to improve service delivery amongst the most vulnerable
- Findings indicate the otological disease progression and associated hearing loss for this hospital cohort is amongst the most severe reported globally
- Patients described numerous barriers to accessing ear care
- Adopting policies that integrate quality ear and hearing care into primary care provision can provide a continuum of care

In order to improve accessibility in remote areas, the use of specialist satellite clinics in isolated areas can also be fruitful.^34^ For example, in the Pacific Islands, co-ordination between developmental organisations, government and local communities helped to establish ear and hearing services in this region by raising awareness and strengthening the collaboration between key stakeholders within the community.^58^ This must be in tandem with developing high quality and affordable services and providing protection against unaffordable and informal costs of accessing care (even within the government system), which remains a challenge in Cambodia despite progress in providing financial protection.^59^

As this was an exploratory study that targeted a selected group, the main limitation was that the experiences and stories collected cannot be seen as representative of the wider community. However, the strength of our study is that we triangulated data using three different methods, providing both breadth and depth in analysing patient care-seeking pathways. Our findings support the need for a rigorous population-based random sample study to further explore the prevalence and burden of ear disease and the regional supply- and demand-side barriers to care. Further analysis of the supply-side barriers to ear and hearing care service delivery would also provide opportunity to understand current clinical management practices and to work with service providers and stakeholders to improve service co-ordination, workforce planning, priority setting, and investment in the ear and hearing care sector.

## Conclusion

This study showed a significant burden of ear disease in Cambodia, made greater by the delay in receiving timely and effective treatment, that exceeds current workforce and infrastructural capacity. The adequate provision of integrated care for non-communicable diseases remains a challenge for the Cambodian health system.^60^ There are apparent gaps in the knowledge of healthcare providers about causes of and treatments for otitis media and a lack of co-ordination of care between providers within the health system. Currently, healthcare providers are unable to impart the information or the management required for appropriate diagnosis and intervention in a timely manner.^61^

The Cambodian Ministry of Health, which delivers government health services at health centres and hospitals, has an ambitious programme for strengthening the availability, quality and affordability of government health services, reflected in the consecutive health strategic plans. Even so, the challenges of integrating ear and hearing care services within existing service-delivery arrangements remain. Three priority ear and hearing care areas for policy makers as they set the agenda under future health strategic plans are: (1) integration of ear and hearing care service delivery into primary care, (2) infrastructure development and human resource training, and (3) sustainable financing and social protection mechanisms in support of ear and hearing care.

Adopting policies that integrate quality ear and hearing care into primary care provision can provide a continuum of care, covering aspects such as health promotion, disease prevention, diagnosis, treatment and disease management through appropriate referral pathways. The global evidence suggests that integrating ear and hearing care within primary care by running patient education campaigns, improving health insurance coverage, investing in provider training and encouraging the take up of mobile health technologies (such as hearing screening) can improve access in rural or remote locations.^62^ This is consistent with the recently published World Report on Hearing, which recommended prioritising a strategy of integrated, community-based and people-centred ear and hearing care to improve local access to interventions and to improve co-ordination of referral pathways.^61^

With regard to the ear and hearing care infrastructure, the improved provision of hearing care by government health facilities could be tackled by strengthening the minimum package of activities and the complementary package of activities delivered at government facilities. This would also serve the purpose of providing patient benefit through existing social health protection mechanisms, such as the health equity funds and newly proposed social insurance arrangements. Health system strengthening in Cambodia in recent years has laid the foundation for moving further in the direction of responding to the emerging burden of non-communicable diseases and in particular for addressing the need for improved hearing care. There is now an opportunity to address more effectively the apparent personal, healthcare and economic burden of hearing loss.

## **Appendix 1.** Patient perspective questionnaire

### Set script (to be translated into Khmer)

As a patient attending the Children's Surgical Centre, you are invited to participate in a questionnaire to explore the barriers people experience in accessing ear and hearing care services in Cambodia. If you choose to participate, you will be asked to complete a 15-minute survey. There are 23 questions, 3 of which are short-answer; all others are multiple choice or tick the box.

Your participation in this research project will in no way affect the care you receive at the Children's Surgical Centre.

Your answers are confidential, and responses are anonymous. We may ask if you would like to be involved in a more in-depth interview if we believe your experience may help provide more information for this study.

Participation is completely voluntary, and you are free to stop at any time while completing the survey. Once you have completed the survey, it is no longer possible to withdraw from the study.

Do you have any questions so far?

(Read plain language statement)

Would you like to participate in this study?

Please sign or provide your thumb print on the consent form which indicates that you agree for your responses to be used for research.

**Table 1.** Patient perspective questionnaire

Part 1: demographics		
What is your gender?		
Female Male Other Prefer not to answer		
What is your age?		
Answer:		
What is your current place of residence? (district, village or town)		
Answer:		
How many hours did you travel to reach the CSC?		
Answer:		
What is the highest education level that you have completed at school?		
Did not attend school Pre-primary school Primary school Secondary school Post-secondary/tertiary		
Employment		
Never worked Currently working Not currently working		
What is your occupation?		
Not currently working Driver Technician Farmer Retired Trader Student Civil servant Other:		
What is your current marital status?		
Never married Currently married Separated Divorced Widowed Cohabiting Do not want to respond		
Part 2: reason for attendance		
What is your reason for today's visit?		
First visit to CSC ENT Department Follow-up visit Ear surgery Post-operative surgical visit Rehabilitation (e.g. hearing aids) Other:		
How did you hear about CSC?		
Health professional referred (e.g. doctor, All Ears Cambodia, other hospital) Family member Friend Social media (e.g. Facebook or Instagram) Radio Other:		
Before coming to CSC, what had been your main ear/hearing symptoms? (circle all that apply)		
Hearing loss Ear pain Discharging or leaking ear Tinnitus (ringing sound or noise in ears) Blocked ear Dizziness or balance problem Other:		
How long did you have these symptoms? (circle closest answer)		
Less than a week Less than a month Up to 6 months Up to 1 year 1–3 years 3–6 years 6–10 years More than 10 years		
What has caused you to seek treatment now? (choose all that apply)		
Worsening symptoms Could not work due to ear problem Recently heard about the services at CSC Other treatments did not work On waiting list at other hospital for too long Change in circumstances (e.g. now living closer to hospital) Referred by health professional Other:		
Part 3: well-being		
What is the MAJOR health problem that limits your activities? (circle only one)		
Diabetes Hypertension/high blood pressure Heart problem Stroke problem Arthritis/rheumatism Back or neck problem Fractures, bone/joint injury Eye/vision problem Ear/hearing problem Lung/breathing problem Cancer Depression/anxiety/emotional problem Other disability or problem (please specify):		
Of each of the following, in the last six months, what effect have your ear problems had on your life?		
a) You have difficulties hearing, even with a hearing aid		
Yes	No	Does not apply
Your ear problem affects your ability to work		
Yes	No	Does not apply
If the ear problem affects ability to work, has lack of money stopped you seeking help?		
Yes	No	Does not apply
Your ear problem affects your ability to enjoy life		
Yes	No	Does not apply
Your ear problem affects your ability to participate in social activities		
Yes	No	Does not apply
Your ear problem affects your ability to take care of your family/household		
Yes	No	Does not apply
Your ear problem affects your ability to keep good family relationships		
Yes	No	Does not apply
Your ear problem affects your ability to have a meaningful life		
Yes	No	Does not apply
Why do you think you have your ear problem?		
Please explain:		
Part 4: access to services		
What services for ear or hearing care are available in your district? (circle all that apply)		
District or referral hospital Private hospital or health centre Qualified doctor or nurse Pharmacy Kru Khmer (traditional healer/herbalist) Monk No services available Other:		
Where you usually live, what are the reasons you could not attend ear care services as much as you needed? (circle all that apply)		
Accessibility No available doctor or service near home No other hospital would do surgery Ineffective referral for ear care Hospital or service was too far away Roads are in poor condition to reach hospital or service No accommodation near hospital or service No one could take care of children Not well enough for surgery at the time On waiting list for too long Knowledge Did not know any ear care services were available Did not think the ear problem was serious enough to need help Did not think anything could be done to help the ear problem Financial I could not miss work Surgery or hearing aid very expensive Transport very expensive Food needed very expensive Childcare expensive Too expensive to bring carer with you to hospital Acceptability Family did not want you to attend the services/facilities Family has difficulty assisting you to access services/facility Belief that the ear problem cannot be fixed by seeing a medical doctor Worry about the treatment or poor outcome of treatment Worry about how people behave towards you at the hospital None Other:		
Among all the reasons you have selected above, which one is the main reason for you?		
(Choose one option) Answer:		
What other ear care services did you seek before coming to CSC? (circle all that apply)		
None District or referral hospital Private hospital or health centre Qualified doctor Specialist ear doctor Pharmacy Drug shop Monk Kru Khmer (traditional doctor/healer) Kru Boramei (fortune teller) Other:		
What other treatment for your ear problem have you received before coming to CSC? (circle all that apply)		
None Medicine from pharmacy Ear surgery Traditional medicine (e.g. herbal remedy) Dietary recommendation Massage Laxatives (banh chos) Bath (toek saoy) or steam bath (chpong) Other (please list):		
*22.* If you could not attend CSC, what other services would you use for your ear problem? (circle all that apply)		
District or referral hospital Other private hospital or health centre Medical doctor Pharmacy Drug shop Monk Kru Khmer (traditional doctor/healer) Kru Boramei (fortune teller) Don't know Other:		
In your community, where do people usually go for their ear problems? (circle all that apply)		
District or referral hospital Private hospital or health centre Qualified doctor Pharmacy Drug shop Monk Kru Khmer (traditional doctor/healer) Kru Boramei (fortune teller) Other: They do not seek help. If they do not seek help, why not?		

CSC = Children's Surgical Centre

You have completed this questionnaire. Thank you for participating in this research project.

## **Appendix 2.** Patient perspective questionnaire: carer

### Set script (to be translated into Khmer)

As you have a child under your care who is a patient attending the Children's Surgical Centre, you are invited to participate in a short questionnaire to find out the problems people have when trying to find help for their ear problems in Cambodia.

If you choose to participate, you will be asked to complete a 15-minute survey. There are 21 questions, 3 of which are short-answer, all others are multiple choice.

Your participation in this research project will in no way affect the care your child will receive at the Children's Surgical Centre.

Your answers are confidential, and responses are anonymous. If you agree, we may contact you later to ask if you would like to come back for a longer interview if we believe your experience may help provide more information for this study.

Participation is completely voluntary, and you are free to stop at any time while completing the survey. Once you have completed the survey, it is no longer possible to withdraw from the study.

If you would like to participate, I will read the plain language statement to ensure you are happy to proceed. Do you have any questions so far?

(Read plain language statement)

Would you like to participate in this study?

Please sign or provide your thumb print on the consent form which indicates that you agree for your responses to be used for research.

**Table 1.** Patient perspective questionnaire: carer

Part 1: demographics		
24. What is the child's gender?		
Female Male Other b) Prefer not to answer		
25. What is the child's age?		
Answer:		
26. Where is the child's current place of residence? (district, village or town)		
Answer:		
27. How many hours did you travel to reach CSC?		
Answer:		
28. What is the highest education level that you, as carer of the child, have completed at school?		
Did not attend school Pre-primary school Primary school Secondary school University Prefer not to answer		
29. Your employment		
Never worked Currently working Not currently working Prefer not to answer		
30. What is your occupation?		
Not currently working Seller Driver Factory or construction worker Teacher Farmer Retired Trader Student Work for the government Other: Prefer not to answer		
Part 2: reason for attendance		
31. What is the reason for the child's visit today?		
First visit Follow-up consultation Ear surgery in-patient Post-operative follow-up consultation Rehabilitation (e.g. hearing aids) Other:		
32. How did you hear about CSC?		
Health professional referred (e.g. doctor, All Ears Cambodia, other hospital) Family member Friend Social media (e.g. Facebook or Instagram) Radio Other:		
33. Before attending CSC, what had been the child's main ear/hearing symptoms? (circle all that apply)		
Hearing loss Ear pain Discharging ear Tinnitus (ringing sound or noise in ears) Blocked ear or ear fullness Dizziness or balance problem Headache Cannot speak Other:		
34. How long did the child have these symptoms from when the problem first began? (circle closest answer)		
Less than a week Less than a month Up to 6 months Up to 1 year 1–3 years 4–6 years 7–10 years 11–15 years More than 15 years		
35. Why did you decide to bring the child to CSC now? (choose all that apply)		
Worsening symptoms Could not go to school due to ear problem Recently heard about the services at CSC Other treatments did not work Other hospital does not treat ear problems Wait too long to have operation at other hospital Change in circumstances (e.g. now living closer to hospital) Referred by health professional Other reason:		
Part 3: wellbeing		
36. In the last six months, what effect have the child's ear problems had on their life?		
a) Difficulties hearing school teacher		
Yes	No	Does not apply
b) Does not attend school because of ear problem		
Yes	No	Does not apply
c) Difficulties talking in family conversations		
Yes	No	Does not apply
d) Difficulties talking with friends		
Yes	No	Does not apply
37. What do you think caused the child's ear problem?		
Please explain:		
Part 4: access to services		
38. What services for ear or hearing care are available in your district? (circle all that apply)		
Government, district or referral hospital or health centre Private hospital or health centre Qualified doctor or nurse Pharmacy Kru Khmer (traditional healer/herbalist) No services available Other:		
39. Do other people in your district use these services		
Yes No. If not, why?		
40. Why did you decide to bring the child to CSC now?		
Accessibility a) Hospital or service was too far away YES / NO b) Roads are in poor condition to reach hospital or service YES / NO c) No one could take care of other children YES / NO d) On waiting list for too long YES / NO Knowledge e) Did not know any ear care services were available YES / NO f) Did not think the ear problem was serious enough to need help YES / NO Financial g) You could not miss work YES / NO h) Treatment was too expensive YES / NO i) Transport very expensive YES / NO j) Food needed very expensive YES / NO k) Childcare expensive for other children YES / NO Acceptability l) Thinks that the ear problem cannot be treated YES / NO m) Felt scared about going to the hospital or service YES / NO If yes, why? n) Any other reason:		
41. Among all the reasons you selected, which one do you believe is the main reason?		
(Choose one option) Answer:		
42. What other ear care services did you seek for the child before coming to CSC? (circle all that apply)		
None Government, district or referral hospital or health centre Private hospital or health centre Qualified doctor Specialist ear doctor Pharmacy Drug shop Kru Khmer (traditional doctor/healer) Other:		
43. What previous treatment for the child's ear problem were sought before coming to CSC? (circle all that apply)		
None Ear drops or topical medicine Oral medication Ear surgery Traditional medicine (eg. herbal remedy) Dietary recommendation Other (please list):		
44. Do you have any other comments or questions?		

CSC = Children's Surgical Centre

You have completed this questionnaire. Thank you for participating in this research project.

## Appendix 3. Topic guide

### Introduction

- Spend time building rapport with participant.
- Remind the participant (and parent or guardian) that this will be audio-recorded and translated into English (then switch on recorders).
- Today, I am interested in what it has been like for you/your child to experience an ear or hearing problem.
- I am also interested in hearing about your journey to finding help for your ear problem.

### Background

Firstly, can you tell me the story of your ear or hearing problem, all the events and experiences that were important for you, up to now?

Prompts:

1. When/how did you first notice your ear problem?
2. What have been your main ear/hearing symptoms?
3. What do you think caused your problem?
4. What do your fear most about your illness?
5. What are the biggest difficulties that your ear problem has caused you? Has the ear problem meant you are unable to work effectively/go to school?
6. How has this problem affected your life up until now? Why do you think it started when it did?
7. How severe is your ear problem? How long do you expect it to last?

### Accessibility/structural

Tell me about what you have done about your ear problem?

Prompts:

1. Where did you go? Are services for ear care available where you live? Tell me about them. Did you use them?
2. How far did you travel?
3. How long did you wait before seeking help for your problem? Why?
4. What has caused you to seek help now?
5. Have there been any problems in accessing ear care services? Tell me about them.

### Affordability/financial

Tell me about what costs you had in coming to find treatment.

Prompts:

1. How were these costs met? E.g. Did you have to borrow money? Does treatment affect the family income? Did you have access to the health equity fund?
2. Direct costs may include: surgery, transport, food, clothes, accommodation for yourself or caregiver, emergency care, informal payments e.g. cost of childcare).
3. Indirect costs (e.g. loss of earnings during surgery/recovery).

### Acceptability/cultural

Tell me about the main concerns you had in coming to a place for care for your ear problem.

Prompts:

1. Did you have some concerns about services in your district?
2. How do other people view hearing problems and ear disease in Cambodia?
3. Did others influence your decision in trying to find help for your ear problem? (significant others, co-workers, teachers, class-mates).
4. What kind of treatment do you think you should receive? What are the most important results you hope to receive from treatment?
5. What do you expect to change into the future?

Thank you for sitting down for this chat with me. Is there anything you would like to add, or do you have any questions?
